# Mapping between headache specific and generic preference-based health-related quality of life measures

**DOI:** 10.1186/s12874-022-01762-y

**Published:** 2022-10-26

**Authors:** Kamran Khan, Hema Mistry, Manjit Matharu, Chloe Norman, Stavros Petrou, Kimberley Stewart, Martin Underwood, Felix Achana

**Affiliations:** 1 Warwick Clinical Trials Unit, Warwick Medical School, University of Warwick, Coventry, CV4 7AL UK; 2 Centre for Health Economics, Warwick Medical School, University of Warwick, Coventry, CV4 7AL UK; 3 University Hospitals Coventry and Warwickshire, Coventry, CV2 2DX UK; 4 Headache Group, Institute of Neurology and The National Hospital for Neurology and Neurosurgery, Queen Square, London, WC1N 3BG UK; 5 Nuffield Department of Primary Care Health Sciences, University of Oxford, Oxford, OX2 6GG UK

**Keywords:** Headache, Migraine, Quality of Life

## Abstract

**Background:**

The Headache Impact Test (HIT-6) and the Chronic Headache Questionnaire (CH-QLQ) measure headache-related quality of life but are not preference-based and therefore cannot be used to generate health utilities for cost-effectiveness analyses. There are currently no established algorithms for mapping between the HIT-6 or CH-QLQ and preference-based health-related quality-of-life measures for chronic headache population.

**Methods:**

We developed algorithms for generating EQ-5D-5L and SF-6D utilities from the HIT-6 and the CHQLQ using both direct and response mapping approaches. A multi-stage model selection process was used to assess the predictive accuracy of the models. The estimated mapping algorithms were derived to generate UK tariffs and was validated using the Chronic Headache Education and Self-management Study (CHESS) trial dataset.

**Results:**

Several models were developed that reasonably accurately predict health utilities in this context. The best performing model for predicting EQ-5D-5L utility scores from the HIT-6 scores was a Censored Least Absolute Deviations (CLAD) (1) model that only included the HIT-6 score as the covariate (mean squared error (MSE) 0.0550). The selected model for CH-QLQ to EQ-5D-5L was the CLAD (3) model that included CH-QLQ summary scores, age, and gender, squared terms and interaction terms as covariates (MSE 0.0583). The best performing model for predicting SF-6D utility scores from the HIT-6 scores was the CLAD (2) model that included the HIT-6 score and age and gender as covariates (MSE 0.0102). The selected model for CH-QLQ to SF-6D was the OLS (2) model that included CH-QLQ summary scores, age, and gender as covariates (MSE 0.0086).

**Conclusion:**

The developed algorithms enable the estimation of EQ-5D-5L and SF-6D utilities from two headache-specific questionnaires where preference-based health-related quality of life data are missing. However, further work is needed to help define the best approach to measuring health utilities in headache studies.

**Supplementary Information:**

The online version contains supplementary material available at 10.1186/s12874-022-01762-y.

## Key points

Several algorithms reasonably predicted health-related quality of life weights from patient responses on headache specific quality of life questionnaires.

New algorithms can be used in cost-effectiveness analyses to guide the money for value assessment of different interventions in different disease areas.

## Background

The Headache Impact Test (HIT-6) [1] and the Chronic Headache Questionnaire (CH-QLQ) [2] (adapted from Migraine Specific Quality of Life version 2 [3] for Chronic Headache disorders) are two measures of headache-related quality of life. Both measures have been validated in patient populations meeting an epidemiological definition of chronic headaches, and shown to have good measurement quality, relevance, and acceptability among headache patients [2]. However, they are not preference-based and cannot therefore be used for the estimation of quality-adjusted life years (QALYs). The QALY is a preference-based measure of health outcome that combines length of life and health-related quality of life (HRQoL) into a single metric and is a standard measure of benefit for economic evaluation purposes. The EuroQoL EQ-5D-5L is the preferred preference-based measure of HRQoL for health care decision makers in many jurisdictions, including in England [4], Wales and Scotland. In headache studies where the HIT-6 or the CH-QLQ but no preference-based measure is collected, a potential solution is to apply a mapping (or ‘crosswalk’) function to convert scores into preference-based (or utility) values. ‘Mapping’ involves the development and use of an algorithm (or algorithms) to predict health-state utility values using data on other indicators or measures of health [5]. The algorithm(s) can be applied to data from clinical trials, observational studies or economic models containing the source predictive measure(s) to predict utility values even though the target preference-based measure was not included in the original source study. The predicted utility values can then be analysed using standard methods for trial-based analyses or summarised for each health state within an economic model [5]. The aim of this study was to develop cross-walk ‘mapping’ algorithms between the HIT-6 or CH-QLQ and generic preference-based measures (EuroQoL EQ-5D-5L [6, 7] and SF-12 version 2 [8]). These algorithms can be used to derive utilities in subsequent analyses, such as economic evaluations reliant on datasets that only include HIT-6 or CH-QLQ information.

## Methods

### Data

To develop the mapping algorithms to generate UK tariffs, a cross-sectional cohort of people living with chronic headaches was recruited from patients who attended headache clinics within NHS hospital outpatient departments in England between September 2019 and March 2020. Patients were eligible to participate if they were aged 18 years and older, had headache symptoms for 15 or more days of the month for at least three months, had good working knowledge of English to understand and complete study questionnaires, were of sound mind and willing and able to give informed consent. Whilst waiting to be seen by their doctor, those meeting the eligibility criteria were informed about the study and given a study information sheet and a consent form. Eligible and consenting participants were asked to complete and return the questionnaire booklet, either before leaving the clinic to a member of the health care team or send it in a prepaid envelope to the study team at the University of Warwick. Demographic and clinical information collected included age and gender, ethnicity, employment status and details of their headaches (number of headache days over a 30-day period, average duration of headache episodes and details of medication use). Outcome data collected included two headache-specific measures (HIT-6 and CH-QLQ) and two generic HRQoL measures (EQ-5D-5L and SF-12 version 2). The SF-12 cannot be used to estimate QALYs directly, instead an algorithm based on the SF-6D allows utility values to be generated from the SF-12 measure [9].

External validation of the mapping algorithms was performed using baseline data from the Chronic Headache Education and Self-management Study (CHESS) randomised controlled trial that compared the clinical and cost-effectiveness of a self-management education support programme plus usual care versus usual care alone for patients with chronic headaches. The CHESS study recruited data on 689 adults aged 18 years and older (356 to the intervention arm and 333 to the control) from primary care and self-referral clinics in London and the Midlands. The external validation dataset consisted of headache-specific (HIT-6 and the CHQLQ v1) and generic HRQoL (EQ-5D-5L and SF-12 v2) questionnaires completed by trial participants at baseline/time of randomisation. The CHESS trial protocol and further details of the trial conduct and population have been reported elsewhere [10].

The study was conducted in accordance with recently published good practice methods and reporting guidelines for estimating health utilities from non–preference-based outcome measures [5]. Written informed consent was obtained from study participants prior to participation in the test and validation sample data collection exercises. For the validation study, ethics approval was provided by the Northwest—Greater Manchester East Research Ethics Committee (REC REF: 16/NW/0890).

### Outcome measures

The HIT-6 is a validated headache-specific measure, whilst the CH-QLQ was adapted from the Migraine Specific Quality of Life Questionnaire V2.1 [11]. The suitability of the CH-QLQ as an outcome measure in studies that recruit people living with chronic headache was evaluated in the CHESS feasibility study. Published analysis of the feasibility data suggests the CH-QLQ has good measurement properties in this population, has greater relevance to the patient experience of chronic headache and is well received by headache patients [12]. The HIT-6 produces a single measure whilst the CH-QLQ reports on three factors; Role Prevention, Role Restriction, and Emotional Function [12]. The EQ-5D-5L and the SF-12 are the most widely used health-related quality of life questionnaires in clinical research. They are preference-based measures which means that they can be converted to health-utilities using established methods [13, 14]. Both cover full range of different recall periods ranging from 1 to 4 weeks and have UK population preference values. In addition, the EQ-5D is also the recommended questionnaire for generating health utilities to inform appraisal of health technologies by NICE [15].

### Statistical analysis

In our analyses, we used direct utility and response mapping approaches to estimate utility scores based on the HIT-6 and CH-QLQ scores. The direct utility approach makes use of regression equations to predict EQ-5D or SF-6D utilities as the dependent variable based on HIT-6 or CH-QLQ scores included in the regression as independent or explanatory variables [5]. The coefficients of the models can then be used to carry out the conversion from the source measure to the target measure in the required datasets. The estimation techniques employed in this paper for the direct utility mapping were: i) Ordinary Least Squares (OLS), ii) Fractional Logistic regression (FLOGIT) [16, 17], iii) Censored Least Absolute Deviations (CLAD) regression [18, 19] and iv) Generalised Linear Modelling (GLM) [20].

To implement FLOGIT, utilities were linearly transformed to obtain a dependent variable bounded between zero and one. Then, a GLM model with a binomial family and a logit link was implemented to predict utility_0-1_. Finally, predictions were back transformed to obtain the usual utility range. We also used response mapping to predict the responses to the SF-6D dimensions as opposed to predicting the summary utility scores directly [21]. A logistic regression model can be used to estimate the probabilities that each set of HIT-6 or CH-QLQ responses corresponds to a response level for each SF-6D dimension. A multinomial logistic model can be used or an ordered logistic model if it is believed that the responses to SF-6D questions are ordered. The models were estimated by fitting a separate multinomial logistic regression (MLOGIT) model for each SF-6D dimension, as described elsewhere [21].

It was not possible to implement direct mapping from the HIT6 or CH-QLQ to the EQ-5D-5L because no published and widely used/acceptable utility tariff currently exists for this instrument in the UK. Instead, we followed the recommendations of a NICE position statement on use of the EQ-5D-5L to map the EQ-5D-5L to the EQ-5D-3L tariff for the UK [14] instead of the published 5L tariff estimated by Devlin et al. (2018) [22]. For these analyses, the utility values are based on cross-walking the 5L to 3L value set, as it was not possible to generate direct response mapping coefficients for the EQ-5D-5L using current methods [15].

For each of the functional forms applied, three sets of covariates were used to predict EQ-5D-5L or SF-6D utility scores. The first set of covariates included the overall HIT-6 score or CH-QLQ sub scale scores (from here on referred to as model 1). The second set included the overall HIT-6 score or CH-QLQ sub scale scores with age and gender as additional covariates (from here onwards referred to as model 2). The third set included the overall scores,, age, gender, and age squared and interaction terms between the overall score for the headache specific-measure and age (from here onwards referred to as model 3). Analyses were conducted in Stata version 17 (Stata-Corp, College Station, TX) [23].

### Assessing model performance

We employed a multi-stage model selection process to short list among the models fitted [24]:

- Step 1: For each regression model, we used the Akaike information criterion (AIC) [25] to determine the best-performing covariate set, and eliminate the other two covariate sets. The lower the AIC, the better the model performance. For models where the AIC could not be calculated (CLAD, MLOGIT), algorithms based on all three covariate sets were selected for inclusion in step two.
- Step 2: For the estimators where the AIC was not available and all three models had been carried forward from step one, we compared the mean squared error (MSE) between models. The model with the lowest MSE in each estimator group/set was carried forward to step three.
- Step 3: Final model selection was based on performance across the range of scores as well as total MSE in the validation sample. To compare the models further, analyses were carried out using results from the validation sample. First, distributions of the predicted and observed utility scores were examined to see how closely predicted values matched observed scores [26]. The proportions of predictions deviating from observed values by < 0.10 or < 0.25 were also calculated to give an indication of the error distribution and how often the models fail to produce useful predictions [27]. For the four selected models, the errors were reported across subsets of the EQ-5D and SF-6D utility score ranges as this is useful for indicating the extend of systematic bias in the predictions [5].

## Results

### Study population

Table 1 presents the summary characteristics of the test and validation samples. Of the 349 patients recruited into the test sample, the overwhelming majority were white (92%), female (82%) and aged between 26 and 55 years (65%). Nearly 60% were in employment, half (54%) left school before the age of 20, 41% left school after age 20 and 4% were attending full-time education. Three percent reported that their headaches usually lasted minutes, half (51%) said their headaches lasted hours and 44% reported that their headaches never went away. The mean number of headache days experienced over the 30-day period preceding assessment was 18.6 (median 20, range: 0 to 30). The median duration of each headache episode among those whose headaches lasted in minutes was 40 min and 8 h among those whose said their headaches lasted hours. 88% reported using medication to alleviate their headache symptoms.

**Table 1:** Summary characteristics of the test and validation study populations

Variable	Level	Test sample, n (%)	Validation sample, n (%)
Total sample	N	349 (100%)	715 (100%)
Gender	Male	61 (17.5%)	120 (16.8%)
	Female	285 (81.7%)	595 (83.2%)
	Other/Unknown	3 (0.9%)	
Age group (years)	18–25	41 (11.7%)	61 (8.5%)
	26–35	66 (18.9%)	109 (15.2%)
	36–45	81 (23.2%)	141 (19.7%)
	46–55	81 (23.2%)	187 (26.2%)
	56–65	54 (15.5%)	129 (18%)
	66–75	21 (6%)	70 (9.8%)
	76–85	2 (0.6%)	16 (2.2%)
	86 +		2 (0.3%)
	Unknown	3 (0.9%)	
Ethnic group	White	321 (92%)	577 (80.7%)
	Black	6 (1.7%)	42 (5.9%)
	Asian	16 (4.6%)	59 (8.3%)
	Mixed	2 (0.6%)	21 (2.9%)
	Unknown	4 (1.1%)	16 (2.2%)
Employment status	Employed	207 (59.3%)	
	Unemployed	6 (1.7%)	
	At school	12 (3.4%)	
	Sickness	68 (19.5%)	
	Family	12 (3.4%)	
	Retired	36 (10.3%)	
	Unknown	8 (2.3%)	
Age left school	None		4 (0.6%)
	Age 12 or less	1 (0.3%)	5 (0.7%)
	Age 13 to 16	90 (25.8%)	170 (23.8%)
	Age 17 to 19	96 (27.5%)	195 (27.3%)
	Age 20 or over	144 (41.3%)	304 (42.5%)
	Full time education	13 (3.7%)	27 (3.8%)
	Unknown	5 (1.4%)	10 (1.4%)
How long headaches last	Minutes	11 (3.2%)	
	Hours	179 (51.3%)	
	Never goes away	153 (43.8%)	
	Unknown	6 (1.7%)	
Medication overuse	No	40 (11.5%)	316 (44.2%)
	Yes	307 (88%)	399 (55.8%)
	Unknown	2 (0.6%)	
Headache type	Definite Chronic Migraine		388 (54.3%)
	Probable Chronic Migraine		327 (45.7%)

Baseline data for 715 patients recruited from primary care practices across London and the Midlands into the CHESS trial served as the validation dataset. The characteristics of these patients mirrored those of the estimation sample. The mean age was 48 years (range 18 to 88), 83% were female and 81% were of white ethnicity. 54% had a definite chronic migraine and 46% probable chronic migraine; 56% reported using medication to alleviate headache symptoms.

### Summary of headache-specific and generic health-related quality of life scores

**Fig. 1:**
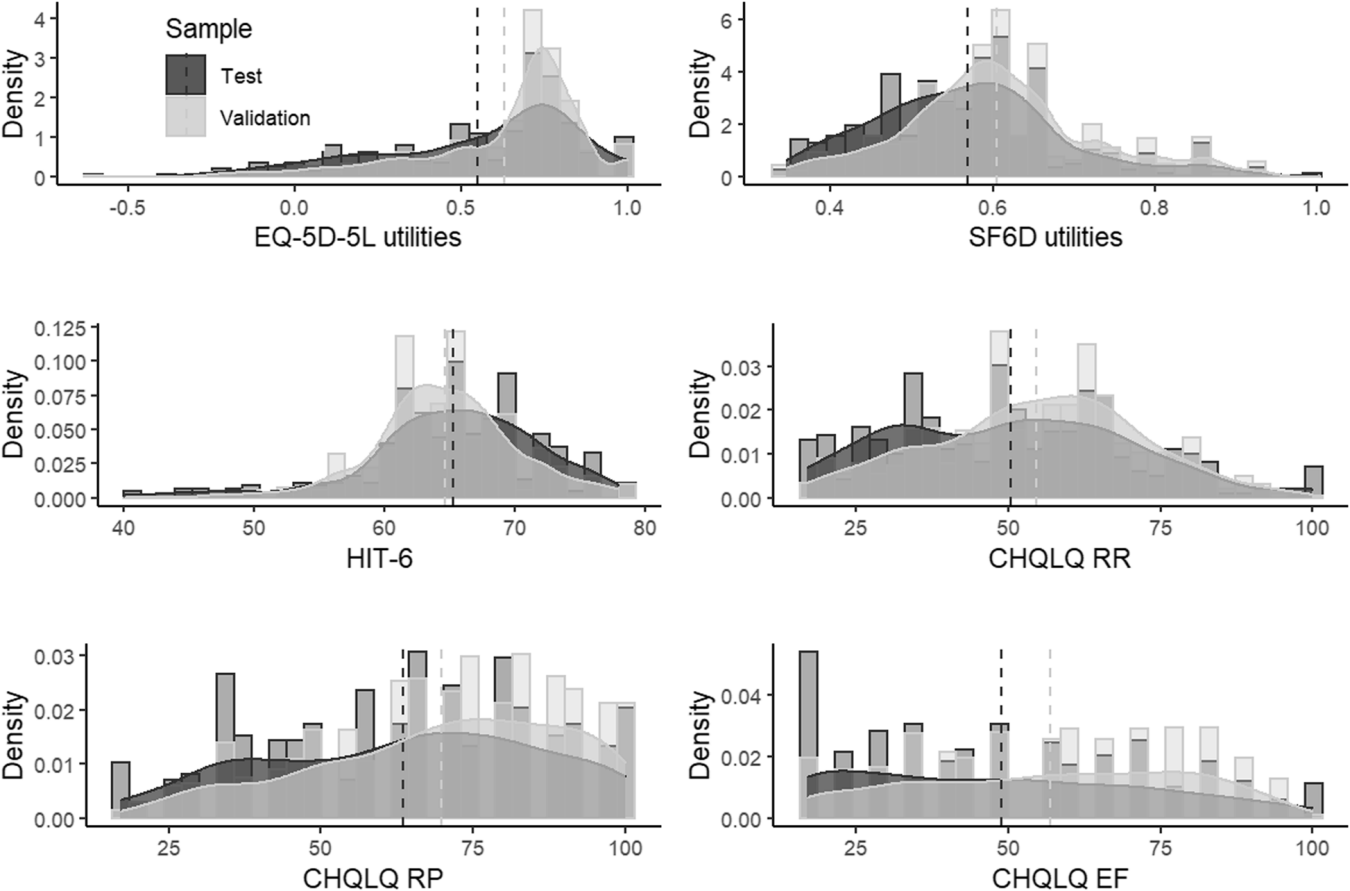
Distribution of outcome measures

Table 2 Broadly in keeping with findings from other patient populations, the EQ-5D utility scores were negatively skewed and bi-modally distributed (Fig. 1) [28]. The SF-6D utility scores, HIT-6 scores and CH-QLQ role function-restrictive scores appeared to be unimodal. The CH-QLQ role function-preventive scores had a right-skewed distribution whereas the CH-QLQ emotional function scores appeared to be left-skewed.

**Table 2:** Summary of headache specific and generic health-related quality of life scores

Outcome	Test Sample (*n* = 349)					Validation Sample (*n* = 715)				
	Mean	Median	Min	Max	SE	Mean	Median	Min	Max	SE
HIT-6	65.25	66	40	78	0.02	64.62	64	42	78	0.01
CHQ-RR	50.31	50	17	100	0.06	54.59	55	17	100	0.02
CHQ-RP	63.42	67	17	100	0.07	69.81	71	17	100	0.03
CHQ-EF	48.79	50	17	100	0.07	56.97	61	17	100	0.03
EQ-5D-5L	0.55	0.65	-0.59	1	0	0.63	0.71	-0.59	1	0
SF-6D	0.57	0.56	0.34	1	0	0.61	0.6	0.34	0.92	0

### Mapping HIT-6 to EQ-5D-5L

Most of the models did not accurately predict the mean EQ-5D-5L to 3L utility score in the validation sample (0.63) with predicted mean EQ-5D utility scores ranging from 0.56 to 0.64; the exceptions were the three CLAD models (Table S1). A difference in utility scores of 0.03 has been externally determined as clinically important for evaluative purposes [29, 30]. No model was able to predict EQ-5D-5L utility scores into the negative range; the model predicting the smallest value for the validation sample was the FLOGIT 3 model (0.09). CLAD 1 and GLM models over predicted the maximum utility score.

### Mapping HIT-6 to SF-6D

All the models accurately predicted the mean SF-6D utility score in the validation sample (0.61) with predicted mean SF-6D utility scores ranging from 0.57 to 0.59 (Table S2). None of the models over predicted the highest SF-6D utility score.

### Mapping CH-QLQ to EQ-5D-5L

Most of the models accurately predicted the mean EQ-5D-5L to 3L utility score in the validation sample (0.63) with predicted mean cross-walked EQ-5D-5L to 3L utility scores ranging from 0.60 to 0.62 (Table S3). Several models were able to predict EQ-5D-5L utility scores into the negative range; the model predicting the largest negative value closest to the actual one for the validation sample was the CLAD 3 model (-0.57). GLM models over predicted the maximum utility score. None of the GLM models with a Gamma family and identity link converged.

### Mapping CH-QLQ to SF-6D

All the models accurately predicted the mean SF-6D utility score in the validation sample (0.61) with predicted mean SF-6D utility scores ranging from 0.58 to 0.60 (Table S4). None of the models over predicted the highest SF-6D utility score.

### Model selection

Models were initially selected based on the AIC or MSE and then filtered by keeping models based on performance across the range of scores as well as total MSE. The selection process resulted in one algorithm being selected for each category of mapping (Table 3). The best performing model for predicting EQ-5D-5L utility scores from the HIT-6 scores was the CLAD (1) model that included the HIT-6 score as the covariate (MSE 0.0550). The selected model for EQ-5D-5L to CH-QLQ was the CLAD (3) model that included CH-QLQ summary scores, age, and gender, squared terms and interaction terms as covariates (MSE 0.0583). The best performing model for predicting SF-6D utility scores from the HIT-6 scores was the CLAD (2) model that included the HIT-6 score and age and gender as the covariates (MSE 0.0102). The selected model for SF-6D to CH-QLQ was the OLS (2) model that included CH-QLQ summary scores and age and gender as covariates (MSE 0.0086).

**Table 3:** Model Performance of selected HIT6 and CH-QLQ to EQ-5D and SF-6D models

			**Predicted EQ-5D Values**								**Abs Diff**	**Abs Diff**
**Model**	**Adj R**^**2**^	**AIC**	**Mean (SD)**	**Min**	**P.25**	**Median**	**P.75**	**Max**	**MSE**	**MAE**	** < 0.10 (%)**	** < 0.25 (%)**
**EQ-5D Models**												
Observed			0.6288 (0.2563)	-0.594	0.531	0.7125	0.7680	1	-	-	-	-
** HIT6 to EQ-5D—CLAD (1)**	0.0929		0.6429 (0.0984)	0.3962	0.5803	0.6540	0.6908	1.0592	0.0550	0.1720	39.51%	78.17%
** CH-QLQ to EQ-5D—CLAD (3)**	0.2933		0.6229 (0.1938)	-0.5704	0.5467	0.6842	0.7582	0.9274	0.0583	0.1702	45.61%	76.83%
**SF-6D Models**												
Observed			0.6056 (0.1163)	0.3450	0.5350	0.6000	0.6600	0.9220	-	-	-	-
** HIT6 to SF-6D—CLAD (2)**	0.2375		0.5926 (0.0649)	0.4292	0.5535	0.5939	0.6297	0.8633	0.0102	0.0751	72.32%	97.25%
** CH-QLQ to SF-6D—OLS (2)**	0.5267	-691.3988	0.5950 (0.0775)	0.4117	0.5393	0.6009	0.6547	0.7544	0.0086	0.0700	75.22%	98.25%

Dependent variable for OLS and CLAD was EQ-5D utility score

Independent variable(s): Model (1) HIT6/CH-QLQ score, Model (2) HIT6/CH-QLQ score, age and gender, Model (3) HIT6/CH-QLQ score, age gender, squared terms, interactions

### Performance of selected models

Table 3 contains the model performance results in the validation sample for the selected models. For each model, in addition to assessing how accurately the models estimated the mean utility scores, we also examined the distributions of the predicted scores. Table 4 shows the MSE and MAE values across the range of utility scores for the selected models. Although the prediction accuracy for the mean scores was similar across models, the level of accuracy was not uniform across the full range of utility scores.

**Table 4:** Distribution of errors by observed EQ-5D and SF-6D range

	HIT-6 CLAD (1)		CH-QLQ CLAD (3)	
EQ-5D Range	MSE	MAE	MSE	MAE
< 0	0.4934	0.6878	0.2794	0.4667
0—0.1	0.2604	0.5020	0.1774	0.3603
0.1—0.2	0.1476	0.3735	0.0671	0.2069
0.2—0.3	0.1139	0.3293	0.1095	0.2899
0.3—0.4	0.0717	0.2566	0.0744	0.2358
0.4 – 0.5	0.0293	0.1479	0.0433	0.1835
0.5 – 0.6	0.0167	0.1086	0.0442	0.1724
0.6 – 0.7	0.0078	0.0676	0.0339	0.1250
0.7 – 0.8	0.0135	0.0985	0.0210	0.0966
0.8 – 0.9	0.0406	0.1824	0.0502	0.1585
0.9 – 1.0	0.1285	0.3300	0.1814	0.3691
	HIT-6 CLAD (2)		CH-QLQ OLS (2)	
SF-6D Range	MSE	MAE	MSE	MAE
0.35 – 0.5	0.0123	0.0937	0.0110	0.0830
0.5 – 0.6	0.0032	0.0451	0.0046	0.0543
0.6 – 0.7	0.0039	0.0499	0.0035	0.0449
0.7 – 0.8	0.0175	0.1165	0.0115	0.0890
0.8 – 0.9	0.0465	0.2045	0.0300	0.1635
0.9—1.0	0.0826	0.2804	0.0665	0.2529

If we first look at the HIT-6 to EQ-5D-5L model, the model was a better predictor towards the upper end of the EQ-5D-5L utility range. For EQ-5D utility scores between 0.4 and 0.8, the model had MSEs between 0.0078 and 0.0293, whereas for predicted values for the remaining range of scores the MSE varied between 0.0406 and 0.1285. The results for the MAEs followed a similar pattern to those for the MSEs. For the CH-QLQ to EQ-5D model, the model was also a better predictor towards the upper end of the EQ-5D utility range and had MSEs between 0.0210 and 0.0442 for EQ-5D utility scores between 0.5 and 0.8.

We now turn to the results for the HIT-6 to SF-6D model. The model was able to accurately predict across most of the range of SF-6D utility scores with MSEs between 0.0032 and 0.0465 for SF-6D utility scores between 0.35 and 0.9. The highest accuracy was observed for SF-6D utility scores between 0.5 and 0.7. The results for the MAEs followed a similar pattern to those for the MSEs. The results for the CH-QLQ to SF-6D model displayed a similar pattern to those of the HIT-6 to SF-6D. Table 5 presents the coefficients for the four selected models. In order to generate utility scores in their own datasets, researchers will need to create any required variables and multiply them by the coefficient values and finally add the constant term.

**Table 5:** Model results

	EQ-5D Models					
Variables	HIT6 to EQ-5D CLAD (1)			CH-QLQ to CLAD (3)		
	Coefficient	Std. Err	P >|t|	Coefficient	Std. Err	P >|z|
HIT6 score	-.0198	0.0075	-	-	-	-
chqrr	-	-	-	0.0107	0.0078	
chqrp	-	-	-	0.0127	0.0078	
chqef	-	-	-	0.0007	0.0070	
chqrr squared	-	-	-	-0.0000	0.0001	
chqrp squared	-	-	-	-0.0001	0.0001	
chqef squared	-	-	-	0.0000	0.0001	
chqrr x chqrp	-	-	-	-0.0000	0.0001	
chqrr x chqef	-	-	-	-0.0002	0.0001	
chqrp x chqef	-	-	-	0.0001	0.0001	
chqrr x age	-	-	-	0.0000	0.0001	
chqrp x age	-	-	-	0.0001	0.0001	
chqef x age	-	-	-	-0.0000	0.0001	
Age	-	-	-	0.0013	0.0099	
Age squared	-	-	-	-0.0001	0.0001	
Sex (Female)	-	-	-	0.0326	0.0350	
Constant	1.9054	0.4764	-	-0.3964	0.3189	
	SF-6D Models					
Variables	HIT6 to SF6D CLAD (2)			CH-QLQ to SF-6D OLS (2)		
	Coefficient	Std. Err	P >|z|	Coefficient	Std. Err	P >|t|
HIT6 score	-0.0118	0.0012	-			
chqrr				0.0018	0.0005	0.001
chqrp				0.0017	0.0004	< 0.001
chqef				0.0009	0.0003	0.012
Age	-0.0002	0.0005	-	-0.0002	0.0003	0.492
Sex (Female)	-0.0142	0.0121	-	-0.0032	0.0124	0.795
Constant	1.3669	0.0919	-	0.3459	0.0313	< 0.001

## Discussion

We present here regression models for mapping between two headache-specific measures (HIT-6 and CH-QLQ) and two generic preference-based health-related quality of life measures (EQ-5D-5L and SF-6D). The performance of the models in terms of overall MAE was similar to previous mapping models, which have obtained MAEs between 0.0011 and 0.19 [31]. The performance in terms of the overall MSE was within the range of other reported studies (0.0071 and 0.0400) for both SF-6D models [31]. In contrast, the overall MSEs for both the EQ-5D models were larger than those of previous studies at 0.0550 and 0.0583.

Model performance in predicting health utilities was variable across the generic and headache specific measures. In general, the models were good at predicting mean EQ-5D-5L and SF-6D utilities with a small error but performed poorly at predicting the tail ends of the utility distribution. None of the models predicted into the negative range of EQ-5D utility scores with the lowest value predicted being 0.09 for the validation sample from the FLOGIT 3 model. Other models generated utilities greater than one, which is the maximum utility possible for people in perfect health.

There are many reasons that could explain why the algorithms developed to predict EQ-5D-5L utilities from responses to the headache specific questionnaires performed particularly poorly. Our mapping functions for the HIT-6 and CH-QLQ produced utilities values with varying levels of precision for the EQ-5D and SF-6D, which may reflect differences between the two utility measures. The SF-6D focuses more on social functioning, while EQ-5D gave more weight to physical functioning, hence the relative contribution of similar domains measuring daily functioning to the utility scores differed substantially [32]. Also, the scoring range of the EQ-5D is much wider (-0.59 to 1) compared with the 0–1 range for SF-6D, this can result in different levels of precisions between the two instruments. In addition, estimating health utilities in chronic headache patients for use in cost-effectiveness analysis is challenging. Patients may only be affected on some days when their health state may be classed as very poor – perhaps for a few hours only. Some standard measures of health utility, such as the EuroQoL EQ-5D questionnaire with a recall period of one-day, assess health status on the day of completion and therefore may not adequately capture the impact of chronic headaches on health-related quality of life if the patient did not have headache on or around the day of questionnaire completion. In contrast, the SF-12 and SF-36, from which SF-6D utilities are derived, the recall period is longer ranging between 1-week and 4-weeks. These instruments may therefore be better at estimating health utilities in patients that have chronic headaches who experience intermittent headaches in some days and no headaches in others. As a consequence, there have been no previous mapping functions between headache specific measures and the EQ-5D-5L, but we are aware of one previous published algorithm to derive a single index of health utility from the 12-Item Short Form Survey (SF-12) [33, 34] and economic evaluations of headache treatments in which health outcomes are expressed in QALY terms [22].

The EQ-5D models developed here are not ideal as they involve double mapping functions to generate utilities from the headache specific outcome measures. This is because there is currently no acceptable UK tariff that can be used for generating health utilities based on the EQ-5D-5L responses for use in economic evaluation studies. The EQ-5D-5L can be converted into health utilities using a recently published value set for England [22]. However, since publication of the EQ-5D-5L value set, NICE has released a position statement [35] advising against the use of the new tariff (13) until the outcome of ongoing research exploring the impact of adopting the EQ-5D-5L valuation set in the NICE reference case becomes available. The position statement further recommends that during this interim period, EQ-5D-5L responses should be mapped or cross-walked onto the EQ-5D-3L using the van Hout et al. [36] algorithm and the health utilities then derived from EQ-5D-3L utility scores using the UK value set for the EQ-5D-3L [14]. Thus, we were only able to carry out direct mappings for the EQ-5D utilities that involve first generating EQ-5D-3L utilities for the EQ-5D-5L responses using the van Hout crosswalk algorithm. The generated mapping coefficients thus involve two embedded mapping functions, and this creates additional level of uncertainty in the algorithms. Further work is needed to help define the best approach to measuring health utilities in headache studies.

## Conclusion

The developed algorithms enable the estimation of EQ-5D-5L and SF-6D utilities from two headache-specific questionnaires where preference-based health-related quality of life data are missing. However, further work is needed to help define the best approach to measuring health utilities in headache studies.

## Supplementary Information

**Additional file 1: Table S1.** Model Performance for HIT-6 to EQ-5D-5L (Estimated using sub study data and validation using CHESS). **Table S2.** Model Performance for HIT-6 to SF-6D (Estimated using sub study data and validation using CHESS). **Table S3.** Model Performance for CH-QLQ to EQ-5D-5L (Estimated using sub study data and validation using CHESS). **Table S4.** Model Performance for CH-QLQ to SF-6D (Estimated using sub study data and validation using CHESS).

## Data Availability

The datasets used and/or analysed during the current study available from the corresponding author on reasonable request.
