# Supplementary material for: Mapping between headache specific and generic preference-based health-related quality of life measures
Source: BMC Med Res Methodol. 2022 Oct 26;22:277. doi: 10.1186/s12874-022-01762-y (PMC9597975; doi:10.1186/s12874-022-01762-y)
Supplement: Supplementary file 1 — Additional file 1: Table S1. Model Performance for HIT-6 to EQ-5D-5L (Estimated using sub study data and validation using CHESS). Table S2. Model Performance for HIT-6 to SF-6D (Estimated using sub study data and validation using CHESS). Table S3. Model Performance for CH-QLQ to EQ-5D-5L (Estimated using sub study data and validation using CHESS). Table S4. Model Performance for CH-QLQ to SF-6D (Estimated using sub study data and validation using CHESS). [file 12874_2022_1762_MOESM1_ESM.docx]

Table S1: Model Performance for HIT-6 to EQ-5D-5L (Estimated using sub study data and validation using CHESS)

| **HIT6** | | | | | | | | | | | | |
| --- | --- | --- | --- | --- | --- | --- | --- | --- | --- | --- | --- | --- |
|  |  |  | **Predicted EQ-5D Values** | | | | | |  |  | **Abs Diff** | **Abs Diff** |
| **Model** | **Adj R^2^** | **AIC** | **Mean (SD)** | **Min** | **P.25** | **Median** | **P.75** | **Max** | **MSE** | **MAE** | **<0.10 (%)** | **<0.25 (%)** |
|  |  |  |  |  |  |  |  |  |  |  |  |  |
| Observed EQ-5D |  |  | 0.6288 (0.2563) | -0.594 | 0.531 | 0.7125 | 0.7680 | 1 | - | - | - | - |
|  |  |  |  |  |  |  |  |  |  |  |  |  |
| **OLS 1** | 0.1233 | 125.89 | 0.5624 (0.0872) | 0.3439 | 0.5069 | 0.5722 | 0.6048 | 0.9310 | 0.0592 | 0.1973 | 25.25% | 74.75% |
| **OLS 2** | 0.1188 | 129.23 | 0.5620 (0.0843) | 0.3179 | 0.5135 | 0.5657 | 0.6119 | 0.8895 | 0.0590 | 0.1974 | 23.82% | 74.32% |
| **OLS 3** | 0.1778 | 108.91 | 0.5819 (0.1155) | 0.1237 | 0.5401 | 0.6117 | 0.6610 | 0.7773 | 0.0563 | 0.1839 | 32.95% | 77.03% |
|  |  |  |  |  |  |  |  |  |  |  |  |  |
| **FLOGIT 1** |  | 291.80 | 0.5671 (0.0924) | 0.3039 | 0.5113 | 0.5839 | 0.6175 | 0.8515 | 0.0579 | 0.1929 | 26.82% | 74.47% |
| **FLOGIT 2** |  | 291.04 | 0.5675 (0.0909) | 0.2681 | 0.5184 | 0.5779 | 0.6254 | 0.8333 | 0.0576 | 0.1927 | 26.11% | 74.18% |
| **FLOGIT 3** |  | 293.83 | 0.5811 (0.1145) | 0.0868 | 0.5487 | 0.6136 | 0.6550 | 0.7510 | 0.0565 | 0.1845 | 31.67% | 77.89% |
|  |  |  |  |  |  |  |  |  |  |  |  |  |
| **CLAD 1** | 0.0929 |  | 0.6429 (0.0984) | 0.3962 | 0.5803 | 0.6540 | 0.6908 | 1.0592 | 0.0550 | 0.1720 | 39.51% | 78.17% |
| **CLAD 2** | 0.1123 |  | 0.6290 (0.0710) | 0.4428 | 0.5887 | 0.6287 | 0.6700 | 0.9413 | 0.0565 | 0.1768 | 37.23% | 78.46% |
| **CLAD 3** | 0.1414 |  | 0.6215 (0.1361) | 0.0973 | 0.5682 | 0.6499 | 0.7144 | 0.8379 | 0.0573 | 0.1753 | 40.09% | 77.03% |
|  |  |  |  |  |  |  |  |  |  |  |  |  |
| **GLM (1) 1** |  | 262.5529 | 0.5626 (0.1123) | 0.3411 | 0.4886 | 0.5642 | 0.6062 | 1.2442 | 0.0605 | 0.1998 | 25.68% | 72.04% |
| **GLM (1) 2** |  | 265.6942 | 0.5620 (0.1067) | 0.3284 | 0.4952 | 0.5563 | 0.6159 | 1.1579 | 0.0599 | 0.1990 | 24.68% | 72.90% |
| **GLM (1) 3** |  | 261.8232 | 0.5850 (0.1242) | 0.2056 | 0.5141 | 0.6076 | 0.6770 | 0.8859 | 0.0568 | 0.1842 | 34.09% | 75.32% |
|  |  |  |  |  |  |  |  |  |  |  |  |  |
| **GLM (2) 1** |  | 257.6750 | 0.5760 (0.1328) | 0.2432 | 0.4916 | 0.5910 | 0.6406 | 1.1374 | 0.0582 | 0.1910 | 28.67% | 73.75% |
| **GLM (2) 2** |  | 261.0204 | 0.5818 (0.1359) | 0.2358 | 0.4996 | 0.5834 | 0.6602 | 1.1506 | 0.0578 | 0.1890 | 30.96% | 73.75% |
| **GLM (2) 3** |  |  |  |  |  |  |  |  |  |  |  |  |
| Dependent variable for OLS, Fractional Logistic, CLAD and GLM was EQ-5D utility score. | | | | | | | | | | | | |
| Independent variable(s): Model (1) HIT6 score, Model (2) HIT6 score, age and gender, Model (3) HIT6 score, HIT6 score squared, age, age squared, gender, interaction term for HIT6 score and age.  (1-Male, 2-Female) GLM (1) model - family(gamma) link(log), GLM (2) model - family(gamma) link(identity) | | | | | | | | | | | | |

Table S2: Model Performance for HIT-6 to SF-6D (Estimated using sub study data and validation using CHESS)

| **HIT6** | | | | | | | | | | | |  |
| --- | --- | --- | --- | --- | --- | --- | --- | --- | --- | --- | --- | --- |
|  |  |  | **Predicted SF-6D Values** | | | | | |  |  | **Abs Diff** | **Abs Diff**  **<0.25 (%)** |
| **Model** | **Adj R^2^** | **AIC** | **Mean (SD)** | **Min** | **P.25** | **Median** | **P.75** | **Max** | **MSE** | **MAE** | **<0.10 (%)** |  |
|  |  |  |  |  |  |  |  |  |  |  |  |  |
| Observed SF-6D |  |  | 0.6056 (0.1163) | 0.3450 | 0.5350 | 0.6000 | 0.6600 | 0.9220 | - | - | - | - |
|  |  |  |  |  |  |  |  |  |  |  |  |  |
| **OLS 1** | 0.3940 | -637.8342 | 0.5771 (0.0603) | 0.4274 | 0.5395 | 0.5843 | 0.6067 | 0.8308 | 0.0108 | 0.0775 | 71.59% | 96.67% |
| **OLS 2** | 0.4001 | -627.3887 | 0.5764 (0.0609) | 0.4174 | 0.5431 | 0.5767 | 0.6122 | 0.8399 | 0.0110 | 0.0782 | 71.59% | 96.52% |
| **OLS 3** | 0.4052 | -627.2439 | 0.5802 (0.0652) | 0.3810 | 0.5459 | 0.5851 | 0.6211 | 0.7969 | 0.0109 | 0.0775 | 72.03% | 96.81 % |
|  |  |  |  |  |  |  |  |  |  |  |  |  |
| **CLAD 1** | 0.2282 |  | 0.5780 (0.0492) | 0.4559 | 0.5473 | 0.5839 | 0.6021 | 0.7850 | 0.0109 | 0.0771 | 71.45% | 96.52% |
| **CLAD 2** | 0.2375 |  | 0.5926 (0.0649) | 0.4292 | 0.5535 | 0.5939 | 0.6297 | 0.8633 | 0.0102 | 0.0751 | 72.32% | 97.25% |
| **CLAD 3** | 0.2473 |  | 0.5807 (0.0630) | 0.3501 | 0.5540 | 0.5956 | 0.6249 | 0.6768 | 0.0114 | 0.0792 | 71.30% | 95.94% |
|  |  |  |  |  |  |  |  |  |  |  |  |  |
| **GLM (1) 1** |  | 292.2385 | 0.5754 (0.0615) | 0.4408 | 0.5359 | 0.5794 | 0.6025 | 0.8904 | 0.0110 | 0.0783 | 71.16% | 96.52% |
| **GLM (1) 2** |  | 292.5761 | 0.5744 (0.0623) | 0.4322 | 0.5395 | 0.5714 | 0.6076 | 0.9041 | 0.0112 | 0.0793 | 71.30% | 96.23% |
| **GLM (1) 3** |  | 298.2999 | 0.5797 (0.0649) | 0.3994 | 0.5431 | 0.5831 | 0.6198 | 0.7970 | 0.0109 | 0.0776 | 72.46% | 96.96% |
|  |  |  |  |  |  |  |  |  |  |  |  |  |
| **GLM (2) 1** |  | 292.0640 | 0.5776 (0.0629) | 0.4214 | 0.5383 | 0.5851 | 0.6085 | 0.8423 | 0.0108 | 0.0776 | 71.59% | 96.67% |
| **GLM (2) 2** |  | 292.4005 | 0.5768 (0.0634) | 0.4111 | 0.5418 | 0.5772 | 0.6137 | 0.8502 | 0.0110 | 0.0784 | 71.59% | 96.52% |
| **GLM (2) 3** |  | 298.3571 | 0.5784 (0.0628) | 0.3982 | 0.5454 | 0.5820 | 0.6169 | 0.8052 | 0.0109 | 0.0778 | 71.74% | 96.52% |
|  |  |  |  |  |  |  |  |  |  |  |  |  |
| **MLOGIT 1** |  |  | 0.5755 (0.0606) | 0.4321 | 0.5355 | 0.5846 | 0.6081 | 0.7887 | 0.0109 | 0.0779 | 72.03% | 96.52% |
| **MLOGIT 2** |  |  | 0.5760 (0.0628) | 0.4235 | 0.5386 | 0.5784 | 0.6167 | 0.8037 | 0.0110 | 0.0781 | 71.74% | 96.52% |
| **MLOGIT 3** |  |  | 0.5776 (0.0638) | 0.4093 | 0.5416 | 0.5805 | 0.6160 | 0.8046 | 0.0110 | 0.0778 | 72.03% | 96.23% |
|  |  |  |  |  |  |  |  |  |  |  |  |  |
| Dependent variable for OLS, GLM and CLAD was SF-6D utility score. Dependent variables for MLOGIT models were SF-6D dimension scores.  Independent variable(s): Model (1) HIT6 score, Model (2) HIT6 score, age and gender, Model (3) HIT6 score, HIT6 score squared, age, age squared, gender, interaction term for HIT6 score and age.  GLM (1) model - family(gamma) link(log), GLM (2) model - family(gamma) link(identity) | | | | | | | | | | | | |

Table S3: Model Performance for CH-QLQ to EQ-5D-5L (Estimated using sub study data and validation using CHESS)

| **Chronic Headache Quality of Life Questionnaire (CH-QLQ)** | | | | | | | | | | | | | |
| --- | --- | --- | --- | --- | --- | --- | --- | --- | --- | --- | --- | --- | --- |
|  |  |  | **Predicted EQ-5D Values** | | | | | |  |  | **Abs Diff** | **Abs Diff** |  |
| **Model** | **Adj R^2^** | **AIC** | **Mean (SD)** | **Min** | **P.25** | **Median** | **P.75** | **Max** | **MSE** | **MAE** | **<0.10 (%)** | **<0.25 (%)** |  |
|  |  |  |  |  |  |  |  |  |  |  |  |  |  |
| Observed EQ-5D |  |  | 0.6288 (0.2563) | -0.594 | 0.531 | 0.7125 | 0.7680 | 1 | - | - | - | - |  |
|  |  |  |  |  |  |  |  |  |  |  |  |  |  |
| **OLS 1** | 0.2895 | 55.0460 | 0.5966 (0.1511) | 0.2109 | 0.4908 | 0.6175 | 0.7196 | 0.8324 | 0.0509 | 0.1708 | 39.42% | 77.55% |  |
| **OLS 2** | 0.3000 | 54.8435 | 0.5984 (0.1546) | 0.1840 | 0.4949 | 0.6237 | 0.7173 | 0.8597 | 0.0503 | 0.1695 | 40.58% | 77.27% |  |
| **OLS 3** | 0.3556 | 36.9935 | 0.6178 (0.1714) | -0.0294 | 0.5392 | 0.6520 | 0.7364 | 0.9912 | 0.0540 | 0.1708 | 42.88% | 77.55% |  |
|  |  |  |  |  |  |  |  |  |  |  |  |  |  |
| **FLOGIT 1** |  | 282.0233 | 0.5986 (0.1463) | 0.1732 | 0.5123 | 0.6314 | 0.7144 | 0.7962 | 0.0500 | 0.1667 | 40.72% | 77.84% |  |
| **FLOGIT 2** |  | 281.6639 | 0.6013 (0.1507) | 0.1375 | 0.5160 | 0.6374 | 0.7172 | 0.8096 | 0.0493 | 0.1649 | 41.29% | 78.13% |  |
| **FLOGIT 3** |  | 297.5530 | 0.6170 (0.1682) | -0.0205 | 0.5462 | 0.6613 | 0.7375 | 0.8955 | 0.0535 | 0.1678 | 44.46% | 78.42% |  |
|  |  |  |  |  |  |  |  |  |  |  |  |  |  |
| **CLAD 1** | 0.2115 |  | 0.6228 (0.1754) | 0.1509 | 0.4995 | 0.6492 | 0.7662 | 0.9075 | 0.0525 | 0.1717 | 38.56% | 77.84% |  |
| **CLAD 2** | 0.2176 |  | 0.6235 (0.1822) | 0.1163 | 0.4962 | 0.6525 | 0.7652 | 0.9543 | 0.0526 | 0.1712 | 40.72% | 77.12% |  |
| **CLAD 3** | 0.2933 |  | 0.6229 (0.1938) | -0.5704 | 0.5467 | 0.6842 | 0.7582 | 0.9274 | 0.0583 | 0.1702 | 45.61% | 76.83% |  |
|  |  |  |  |  |  |  |  |  |  |  |  |  |  |
| **GLM (1) 1** |  | -112.7963 | 0.6083 (0.1984) | 0.2233 | 0.4450 | 0.6027 | 0.7657 | 1.0512 | 0.0574 | 0.1874 | 33.38% | 73.38% |  |
| **GLM (1) 2** |  | -232.7785 | 0.6098 (0.2039) | 0.1969 | 0.4528 | 0.6022 | 0.7571 | 1.0697 | 0.0578 | 0.1878 | 33.81% | 73.96% |  |
| **GLM (1) 3** |  | - | NA | - | - | - | - | - | - | - | - | - |  |
|  |  |  |  |  |  |  |  |  |  |  |  |  |  |
| **GLM (2) 1** |  |  | NA |  |  |  |  |  |  |  |  |  |  |
| **GLM (2) 2** |  |  | NA |  |  |  |  |  |  |  |  |  |  |
| **GLM (2) 3** |  |  | NA |  |  |  |  |  |  |  |  |  |  |
| Dependent variable for OLS, Fractional Logistic, CLAD and GLM was EQ-5D utility score. | | | | | | | | | | | | | |
| Independent variable(s): Model (1) CH-QLQ summary scores, Model (2) addition of age and gender, Model (3) squared terms and interaction terms  GLM (1) model - family(gamma) link(log), GLM (2) model - family(gamma) link(identity) | | | | | | | | | | | | | |

Table S4: Model Performance for CH-QLQ to SF-6D (Estimated using sub study data and validation using CHESS)

| **Chronic Headache Quality of Life Questionnaire (CH-QLQ)** | | | | | | | | | | | | | |
| --- | --- | --- | --- | --- | --- | --- | --- | --- | --- | --- | --- | --- | --- |
|  |  |  | **Predicted SF-6D Values** | | | | | |  |  | **Abs Diff** | **Abs Diff** |  |
| **Model** | **Adj R^2^** | **AIC** | **Mean (SD)** | **Min** | **P.25** | **Median** | **P.75** | **Max** | **MSE** | **MAE** | **<0.10 (%)** | **<0.25 (%)** |  |
|  |  |  |  |  |  |  |  |  |  |  |  |  |  |
| Observed SF-6D |  |  | 0.6056 (0.1163) | 0.3450 | 0.5350 | 0.6000 | 0.6600 | 0.9220 | - | - | - | - |  |
|  |  |  |  |  |  |  |  |  |  |  |  |  |  |
| **OLS 1** | 0.5229 | -700.6506 | 0.5944 (0.0775) | 0.4079 | 0.5381 | 0.6024 | 0.6549 | 0.7545 | 0.0086 | 0.0700 | 75.07% | 98.25% |  |
| **OLS 2** | 0.5267 | -691.3988 | 0.5950 (0.0775) | 0.4117 | 0.5393 | 0.6009 | 0.6547 | 0.7544 | 0.0086 | 0.0700 | 75.22% | 98.25% |  |
| **OLS 3** | 0.5202 | -677.3101 | 0.5958 (0.0810) | 0.4107 | 0.5341 | 0.5990 | 0.6564 | 0.7647 | 0.0087 | 0.0708 | 75.66% | 98.40% |  |
|  |  |  |  |  |  |  |  |  |  |  |  |  |  |
| **CLAD 1** | 0.3194 |  | 0.5786 (0.0630) | 0.4252 | 0.5357 | 0.5865 | 0.6234 | 0.7129 | 0.0093 | 0.0716 | 74.93% | 97.67% |  |
| **CLAD 2** | 0.3250 |  | 0.5932 (0.0740) | 0.4208 | 0.5391 | 0.5966 | 0.6447 | 0.7768 | 0.0087 | 0.0703 | 75.80% | 98.25% |  |
| **CLAD 3** | 0.3377 |  | 0.5977 (0.0838) | 0.2806 | 0.5462 | 0.6118 | 0.6613 | 0.8063 | 0.0098 | 0.0756 | 71.72% | 97.96% |  |
|  |  |  |  |  |  |  |  |  |  |  |  |  |  |
| **GLM (1) 1** |  | 288.6618 | 0.5933 (0.0796) | 0.4214 | 0.5342 | 0.5955 | 0.6530 | 0.7761 | 0.0086 | 0.0701 | 76.09% | 98.40% |  |
| **GLM (1) 2** |  | 290.0222 | 0.5935 (0.0793) | 0.4252 | 0.5336 | 0.5952 | 0.6520 | 0.7729 | 0.0086 | 0.0701 | 75.22% | 98.25% |  |
| **GLM (1) 3** |  | 309.9263 | 0.5954 (0.0806) | 0.4174 | 0.5345 | 0.5985 | 0.6546 | 0.7637 | 0.0087 | 0.0708 | 75.22% | 98.40% |  |
|  |  |  |  |  |  |  |  |  |  |  |  |  |  |
| **GLM (2) 1** |  | 288.6885 | 0.5938 (0.0761) | 0.4105 | 0.5388 | 0.6017 | 0.6533 | 0.7510 | 0.0086 | 0.0699 | 75.51% | 98.25% |  |
| **GLM (2) 2** |  | 290.0525 | 0.5943 (0.0759) | 0.4156 | 0.5407 | 0.6012 | 0.6529 | 0.7487 | 0.0086 | 0.0699 | 75.22% | 98.25% |  |
| **GLM (2) 3** |  | 309.9223 | 0.5952 (0.0800) | 0.4156 | 0.5351 | 0.5996 | 0.6540 | 0.7631 | 0.0087 | 0.0709 | 74.64% | 98.40% |  |
|  |  |  |  |  |  |  |  |  |  |  |  |  |  |
| **MLOGIT 1** |  |  | 0.5904 (0.0770) | 0.4190 | 0.5346 | 0.5946 | 0.6505 | 0.7620 | 0.0086 | 0.0700 | 75.36% | 98.40% |  |
| **MLOGIT 2** |  |  | 0.5919 (0.0783) | 0.4242 | 0.5346 | 0.5915 | 0.6507 | 0.7843 | 0.0086 | 0.0699 | 74.78% | 98.54% |  |
| **MLOGIT 3** |  |  | 0.5939 (0.0774) | 0.4073 | 0.5436 | 0.5919 | 0.6465 | 0.7722 | 0.0089 | 0.0709 | 75.07% | 98.25% |  |
| Dependent variable for OLS, GLM and CLAD was SF-6D utility score. Dependent variables for MLOGIT models were SF-6D dimension scores. | | | | | | | | | | | | | |
| Independent variable(s): Model (1) CH-QLQ summary scores, Model (2) addition of age and gender, Model (3) squared terms and interaction terms  GLM (1) model - family(gamma) link(log), GLM (2) model - family(gamma) link(identity) | | | | | | | | | | | | | |
